# IoT-Forensics Meets Privacy: Towards Cooperative Digital Investigations

**DOI:** 10.3390/s18020492

**Published:** 2018-02-07

**Authors:** Ana Nieto, Ruben Rios, Javier Lopez

**Affiliations:** Network, Information and Computer Security (NICS) Lab, University of Malaga, 29071 Malaga, Spain

**Keywords:** IoT-forensics, digital witness, privacy

## Abstract

IoT-Forensics is a novel paradigm for the acquisition of electronic evidence whose operation is conditioned by the peculiarities of the Internet of Things (IoT) context. As a branch of computer forensics, this discipline respects the most basic forensic principles of preservation, traceability, documentation, and authorization. The digital witness approach also promotes such principles in the context of the IoT while allowing personal devices to cooperate in digital investigations by voluntarily providing electronic evidence to the authorities. However, this solution is highly dependent on the willingness of citizens to collaborate and they may be reluctant to do so if the sensitive information within their personal devices is not sufficiently protected when shared with the investigators. In this paper, we provide the digital witness approach with a methodology that enables citizens to share their data with some privacy guarantees. We apply the PRoFIT methodology, originally defined for IoT-Forensics environments, to the digital witness approach in order to unleash its full potential. Finally, we show the feasibility of a PRoFIT-compliant digital witness with two use cases.

## 1. Introduction

Computer forensics is in constant evolution. This discipline is incessantly adapting its tools, procedures and methodologies to cover new contexts and scenarios. Such is the case of IoT-Forensics [1], which is the term coined to describe a new branch of computer forensics dedicated to the particular features and requirements of digital investigations in Internet of Things (IoT) scenarios.

**Figure 1:**
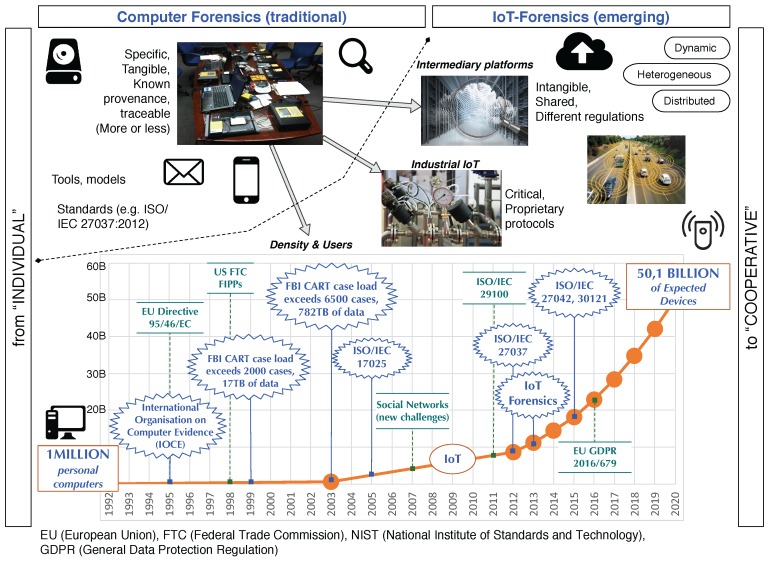
From computer forensics to IoT-Forensics.

The adaptation of computer forensics to take into account IoT scenarios is necessary due to a number of characteristics that make forensic analysis in the IoT different from other contexts or paradigms. Existing computer forensic branches cannot be successfully applied to the new features and requirements imposed by the IoT, namely:

- Increasingly greater numbers of devices (see Figure 1)
- Greater heterogeneity of devices that call for specialized information-retrieval tools
- Widespread implementation of proprietary protocols
- Massive amount and diversity of collected data, complicating the identification of relevant data
- Need for new formats to store digital evidence in IoT devices
- Existence of numerous resource-constrained devices
- Ubiquitous deployment of context-aware devices

These challenging features have resulted in a substantial effort being made towards the definition and development of digital forensic solutions in the context of the IoT paradigm (c.f. Section 5). Despite these efforts, most computer forensics solutions have so far neglected the need for protecting individual privacy throughout digital investigations. This is true even though IoT devices are known to be capable of collecting and storing vast amounts of personal information as they become an integral part of our lives. Not only are smartphones used and deployed among individuals but also wearables, smart implantable gadgets, and countless sorts of context-aware devices. Consequently, we strongly believe that the hefty gap between computer forensics and privacy needs to be narrowed.

Traditional digital forensic mechanisms and tools, such as those used for the seizure of digital evidence at a crime scene, are prepared for static contexts, in which the voluntary participation of citizens is not required. In such scenarios, the concept of *witness* is applied to individuals, not to devices, or tools. In highly dynamic scenarios, like those envisioned by the IoT, the acquisition of digital evidence is much more complex and it may be crucial for the investigator to get help from nearby citizens and devices. Without a cooperative approach it is very difficult to understand the whole context, since the information can be distributed and volatile information could otherwise be lost. This is where the concept of digital witness comes into play.

A *digital witness* [2] is a novel solution to obtain digital evidence in IoT scenarios. However, as shown in Section 3.1, this solution has some serious privacy limitations. Although some mechanisms can be applied to mitigate these problems [3], privacy requirements are not considered throughout the whole lifecycle of this solution. Only when privacy is guaranteed at all times will it be possible to deliver a solution for obtaining digital evidence in the IoT driven by the cooperation of witnesses. It is, therefore, necessary to establish the privacy principles that should enforced in each of the phases of the digital witness approach.

The PRoFIT (*Privacy-aware IoT-Forensics*) model was defined in [4] to integrate privacy properties in accordance with ISO/IEC 29100:2011 [5] throughout the various phases of a forensics model adapted to the IoT. Unlike previous approaches, PRoFIT highlights the importance of collaborating with nearby devices to gather electronic evidence that helps to fully clarify the context of a crime scene. In fact, this generic IoT-based computer forensics model neatly fits the digital witness concept.

In this paper, the digital witness solution is adapted to make it compliant with the PRoFIT model. Therefore, a series of standard privacy principles are considered as an integral part of the lifecycle of the digital witness solution. This is expected to boost the cooperation of citizens in digital investigations carried out in dynamic IoT environments as they can trust the way in which the system handles the potentially sensitive information stored in their personal devices.

This paper is organised as follows. Section 2 describes the fundamentals of both the *digital witness* approach and the PRoFIT model. Then, Section 3 details the specific improvements achieved by adapting the digital witness approach to the PRoFIT model and presents the characteristics of the PRoFIT-compliant digital witness. Section 4 presents two use cases to analyse the PRoFIT-based DW methodology. Next, Section 5 discusses the related work. Finally, Section 6 presents the conclusions of this paper and outlines potential lines of future work.

## 2. Background

This section provides an overview of the concepts and proposals which pave the way towards a privacy-respectful solution for conducting digital forensics investigations in the context of the Internet of Things. First, we introduce the notion and approach of digital witnesses and then we review the methodology of the PRoFIT model together with the privacy needs identified for each phase of the methodology.

### 2.1. Digital Witness

A *digital witness* (DW) is a personal device capable of identifying, collecting, safeguarding and communicating digital evidence [2]. This digital evidence can be used to substantiate a criminal investigation since the very nature of digital witnesses ensure that the evidence is protected and has not been tampered with at any time. In fact, the main goal of the digital witness approach is to deploy what we call the *Digital Chain of Custody in the IoT* (DCoC-IoT), which is a more practical and flexible way of implementing a digital chain of custody using personal devices while retaining the integrity and traceability guarantees offered by a traditional chain of custody.

To operate as a digital witness and be eligible as a member of a DCoC-IoT, a personal device must satisfy the following properties or capabilities:

- *Anti-tampering behavior*: a digital witness must integrate some form of *Trusted Computing Hardware* (e.g., Secure Element, TPM) to provide anti-tampering capabilities to the device. Embedding such hardware inside the device enables not only detecting whether digital evidence has been compromised but also performing periodic integrity checks of the device. If the device is found to have been corrupted it will not be allowed to participate in a DCoC-IoT.
- *Binding credentials*: a digital witness must be unequivocally bound to the identity of its owner or the entity who is responsible for the device. The ultimate goal of electronically binding digital evidence to a device, which in turn operates *on behalf of its owner*, is to dissuade people from misusing digital witnesses to report fake evidence, and helps to implement the traceability of the digital evidence during the process of the binding delegation.
- *Binding delegation*: a digital witness must be allowed to transmit digital evidence to other authorised digital witnesses to enable the deployment of the DCoC-IoT. The way in which the DCoC-IoT is deployed depends on the roles and capabilities of the devices. Some devices may play the role of digital witness but others can also play the role of digital custodian. Digital custodians are owned by agents of the law and are thus usually preferable when deploying the DCoC-IoT.
- *Well-defined procedures*: a digital witness must behave according to a set of well-defined procedures and standards for compliance with the digital evidence management process. This includes a number of established phases and robust cryptographic mechanisms that guarantees the integrity both of the evidence and the DCoC-IoT.

Once digital evidence has been collected by a digital witness, tit is sent, possibly using other digital witnesses (or custodians), toward the *Official Collection Point* (OCP). The OCP is the final link in the DCoC-IoT and is in charge of analysing and processing the digital evidence following the processes established by the ISO/IEC 27042:2015 [6] standard. However, neither the processes described in this standard nor other existing solutions devised for the management of digital evidence in the IoT (c.f. Section 5) consider the collaboration of personal devices. The collaboration principle is indeed core to the digital witness approach but unfortunately there is no direct map between the phases of the ISO/IEC standard and those considered by the digital witness.

**Figure 2:**
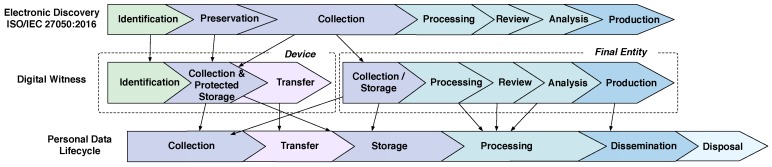
Data lifecycle and its relationship with ISO/IEC 27050:2016 and Digital Witness.

This problem is solved by separating the phases of the digital witness approach into two blocks, which are based on the main actor involved in the process (see Figure 2). In the first block, the personal device is in charge of the identification, preservation (within the device) and transfer of digital evidence, while the second block involves the collection (from multiple sources), processing, review, analysis and production of results by the OCP. Figure 2 shows the mapping between the phases of a digital witness approach and the phases of data lifecycle [7].

The ISO/IEC 27050:2016 standard defines phases to handle Electronically Stored Information (ESI), which are similar to the desired phases for a digital evidence management process (c.f. Figure 2). Note that, unlike ISO/IEC 27050:2016, the digital witness approach considers privacy to be of the utmost importance as people might be reluctant to collaborate in an investigation if they feel their privacy is at stake by sharing data within their personal devices. Data privacy is not considered by the ISO/IEC standard most probably because it assumes that personal data are not transferred but rather collected by an authorised expert and not shared with other entities. However, our approach is more flexible in this particular regard, yet still privacy-preserving and secure.

Note that the original definition of the digital witness scheme [2] considers the need for *privacy policies*, however, these are intended to (i) allow the owner of the device to choose which type of data can be collected, and (ii) ensure that the user is well aware of and accepts the terms of the service being provided. Clearly, these policies are not sufficient to solve the privacy problems identified in [3]. In particular, two major problems are:

- The digital witness approach allows other devices in the environment - and not just the OCP and authorised digital witness—to obtain information about users who were not even directly related to the offence. This is because the digital witness collects information from the surroundings of the digital device without asking for third-party consent beforehand.
- The data collected by a digital witness could be used to infer sensitive information which is not relevant to the investigation.

Therefore, it is not enough to ensure users’ consent about their own data, or the admissibility of the evidence, but also to protect the rights of third parties.

Moreover, some user privacy requirements may be in direct conflict with the requirements that enable a personal device to be a digital witness, for example, anonymity and binding credentials. In this paper we extend the privacy capabilities of the digital witness approach with PRoFIT.

### 2.2. The PRoFIT Model

The Privacy-aware IoT-Forensics (PRoFIT) model is presented in [4] as a mechanism to stimulate the cooperation of citizens in digital forensics investigations. The proposed model takes into consideration a series of privacy principles and applies them throughout the lifecycle of personal data in order to allow citizens to retain control of the sensitive information stored in their personal IoT devices while collaborating with an investigation.

When users are comfortable with the data collection and processing practices offered by PRoFIT, they are willing to share their data with forensics investigators. A direct consequence of this is that digital investigations can be carried out in a more timely fashion without the hassle of asking for warrants or causing inconvenience to citizens, who are no longer forced to hand over their personal devices for an indeterminate length of time, until the investigation has come to an end.

#### 2.2.1. Methodology

**Figure 3:**
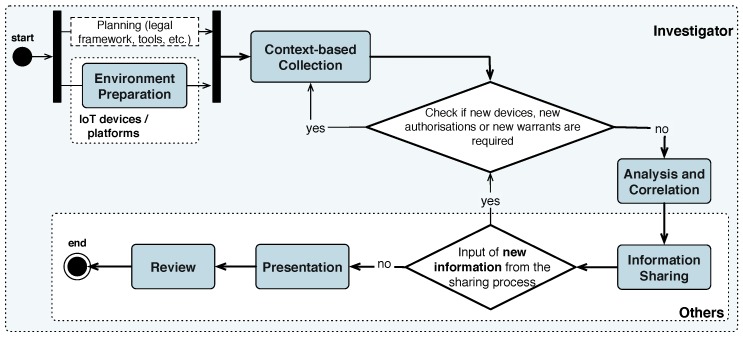
The PRoFIT model [4].

A general overview of PRoFIT is given in Figure 3. The various phases of the model can be classified according to the stakeholders involved in them, namely, the citizens and their devices, the investigator/analyst, and other actors who are either not directly related to the analysis or participate in the last phases of the digital investigation (e.g., an external analyst, a lawyer or a judge who reads the report, etc.):

- *Citizens*: the first phase gives citizens the opportunity to prepare their own personal devices. The preparation of devices consists of installing the PRoFIT software to assist citizens in future privacy- and forensics-related decisions. This phase is accomplished before the start of the investigation. Note that citizens are not forced to perform these actions but are encouraged to do so since the installation of this software will aid them in subsequent phases of the investigation.
- *Investigator*: the involvement of the investigator spans the first three phases of the methodology. In the first phase, the investigator performs all actions necessary for the preparation of a case, namely, the analysis of the legal framework, configuration of data forensic tools, etc. The second phase is devoted to the collection of data from the IoT devices either from citizens willing to collaborate or, in the worst case, obtained through a warrant. In the third phase, the investigator analyses and correlates the data obtained from the previous phase.
- *Other actors*: the results of the analysis performed in the previous phases are prepared to be presented to non-technical stakeholders involved in the digital investigation (e.g., a client) who requested the investigation, a court of law, etc.). Moreover, this includes the processes related to the release of the digital evidence and the return of artefacts to their corresponding owners.

The PRoFIT methodology first asks non-personal devices for the information they collected. Only when these devices cannot provide sufficient evidence to settle an investigation, will personal devices be queried. Notwithstanding, non-personal devices may also belong to, or be managed by, one or more individuals, or organisations handling sensitive information. As such, the investigator will need to get permission from those responsible for the device in order to gain access to the data it holds. In any case, these data are less likely to be privacy-sensitive compared to data within personal devices.

More details about the phases and methodology of the PRoFIT model can be found in [4].

#### 2.2.2. Privacy Principles

PRoFIT integrates privacy-respectful practices as part of the lifecycle of digital investigations. In particular, PRoFIT applies the 11 privacy principles defined by the ISO/IEC 29100:2011 standard [5] to the various phases of the methodology described in the previous section.

These privacy principles are concerned with providing citizens more control over who can access their data, to what extent, and for what purpose. They also make the data handler responsible for protecting these data from unauthorised access, loss and manipulation, as the data handler is liable for any leaks or harm caused to the data. Table 1 summarises how PRoFIT integrates such principles in the phases of the model. Basically, in the initial phases the methodology focuses on privacy principles related to citizens’ awareness and data minimisation. After data collection, the model mainly concentrates on keeping data away from unauthorised entities and uses. During the last phases of the investigation, special attention is paid to principles related to data quality and limitation of use, retention and disclosure. Finally, note that PRoFIT enforces the application of audits and procedures throughout the whole process to ensure the privacy compliance of the system.

Note that the upcoming General Data Protection Regulation (GDPR) is a game changer. However, it is not clear to us whether it will have a strong impact on computer forensics as we know them today. For that reason, the PRoFIT methodology tries to lay the foundations for voluntary data sharing based on trust where the data handler will follow a set of privacy-respectful practices (i.e., privacy principles). Without trust, citizens are reluctant to collaborate.

As for the aforementioned privacy principles (ISO/IEC 29100), they are not exactly the same as those proposed by GDPR but there is an easy mapping between them. In fact, most existing data protection regulations, directives and standards are based on the Organisation for Economic Co-operation and Development (OECD) privacy principles from 1980 with some minor nuances. All things considered, PRoFIT provides citizens with sufficient guarantees that the data they share with the system are collected for a particular investigation and will not be used for other purposes or shared with third parties. Data will be kept safe at all times and the citizen has the right to access them and even withdraw them from offer. By affording such a level of control to citizens, the system becomes more trustworthy and in turn citizens feel more disposed to collaborate with investigations by sharing their data.

## 3. PRoFIT-Compliant Digital Witness

This section shows how PRoFIT can be applied to the scenario of digital witnessing. First, we show how the mitigation mechanisms identified in [3] can be mapped to the PRoFIT phases. Subsequently, we describe how the digital witness architecture must be adapted to be *PRoFIT-compliant* and thus support the first phase of the model (c.f. Section 2.2.1), which consists of the preparation of the devices.

### 3.1. Mitigation of Privacy Risks

As discussed in [3], the original digital witness approach posed several privacy risks. These risks are summarised in Table 2 together with some mechanisms that can be applied to mitigate them. These mitigation mechanisms were suggested in the aforementioned paper. Table 2 also shows that the mitigation mechanisms can be applied at different phases of a PRoFIT-compliant digital witness solution.

**Figure 4:**
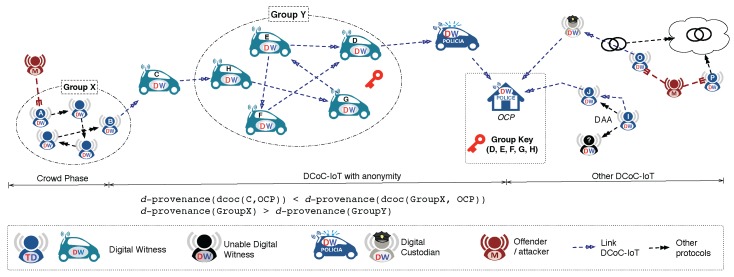
Anonymous digital witnessing.

In other words, a PRoFIT-compliant digital witness meets the privacy requirements of the ISO/IEC 29100:2011 standard (c.f. Table 1), but also allows for the implementation of the following mitigation mechanisms:

M1. *Direct Anonymous Attestation*. Allows a verifier to check whether a platform uses a certified hardware security module without revealing the identity of the platform’s user.
M2. *Blind signatures plus signature chaining*. These mechanisms can be used to certify that a given piece of evidence existed at time *T* without revealing its actual contents to the signer.
M3. *Homomorphic encryption or secure computation*. The witnesses can collaboratively share and operate on the statements of other participants without learning the contributions of each other.
M4. *Anonymous Digital Witnessing*. This solution relaxes the definition of digital witness to enable digital evidence to be reported without revealing the real identity of the source. For example, a set of digital witnesses could use a Crowds-like anonymous communication system to report digital evidence without identifying the precise digital witness which requested the investigation. As detailed in [3] and shown in Figure 4, this solution does not provide *full anonymity*, as this is not allowed because it would affect the traceability requirement (c.f. binding credentials in Section 2.1). Instead, it offers a solution in which i) anonymity is provided at source (defining the concept of d-provenance largely discussed in [3]) but ii) the links in the chain of custody can only opt for the option of anonymity if it is revocable by the OCP.
M5. *Anonymous route discovery*. Enables the discovery of routes to the OCP without revealing the identity of the initiator. This can be achieved by adapting protocols such as the *Authenticated Anonymous Secure Routing* (AASR) to the digital witness solution.
M6. *Third-party user consents*. Not only the user of the digital witness, but all the witnesses involved in a collaboration, accept the policies under a specific context.
M7. *Privacy-aware smart contracts*. Avoid non-repudiation of transactions while maintaining privacy.
M8. *Disposal guarantees*. A verifier can check by means of proof of secure erasure whether or not a third party has erased some particular portions of its memory.

Mitigations M1–M4 must be addressed during the preparation phase because they require the digital witnesses to be ready for handling these operations (e.g., holding suitable cryptographic material). Mitigation M4 also affects the analysis and correlation phase because the approach should ensure that the identity of source witnesses cannot be known after the correlation of the data. The same occurs with M5; route discovery could leak information about third parties when anonymity is not considered during the *binding delegation* process. However, in this case, it could leak information on actors who were on the scene but did not participate in the transaction.

Moreover, the solution must ask for third-party user consents (M6) during context-based collection as consents must be checked during the rest of the phases in different ways. This not only means to inform the user (which is already considered in [2]), but also to be able to obtain the consent of the rest of the collaborators (i.e., witnesses). Authorisations must be checked throughout the whole process.

Mitigation M7 serves the purpose of ensuring that data transactions not only guarantee non-repudiation but also allow the introduction of transactional privacy [12]. Therefore, this mitigation mechanism could be applied not only during the collection phase but also in the process of sharing information between different entities and, finally, in the reviewing phase to verify the correct execution of transactions. In addition, note that in Table 2 all the mitigation mechanisms affect the context-based collection phase. The reason for this is that it is the first phase where a digital witness encounters third-party data, therefore this is an important characteristic that affects the privacy principles in this solution.

Finally, disposal guarantees (M8) must be ensured to all the users during the collection phase and must be implemented once the review phase has taken place.

It is important to clarify that the list of mitigation mechanisms presented in this section is not exhaustive. Our goal is simply to showcase already existing mechanisms that can cope with the privacy issues arising in the context of digital witnessing, and how they can be mapped into the different phases of the methodology. Clearly, more techniques exist and more will appear in the future, which one to use will depend on the features and requirements of the platform at the time of implementing the mitigation mechanism.

### 3.2. PRoFIT Privacy Manager Component (PPM) for Digital Witness

**Figure 5:**
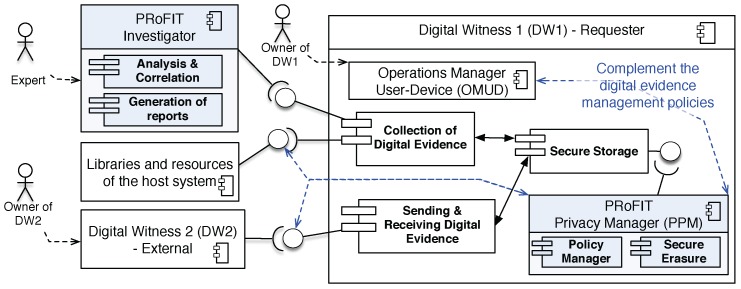
PRoFIT-compliant Digital Witness architecture.

Figure 5 shows the architecture of a digital witness that requests the start of a digital forensics investigation (DW1, right), which will be conducted by the PRoFIT investigator (left). Note that the PRoFIT investigator acts as OCP (Section 2.1). The forensic investigation may also require other digital witnesses to cooperate, which has been denoted as an external digital witness (DW2). DW2 has the same components as DW1.

The original definition of digital witness [2] considered two groups of policies (c.f. Section 2.1). The first group (GP1) defines the user policies within the Operations Manager User-Device (OMUD), and the second group (GP2) defines policies for digital evidence management according to existing standards. This paper focuses on the first group of policies because they are directly related to the decisions made by the user about the data that his/her device will collect, and, therefore, affects privacy. The second set of policies deals with protecting such information once it is available, and to follow standard procedures that are not under the user’s control.

Therefore, the architecture of a digital witness (right of Figure 5) can be adapted to be PRoFIT-compliant, as follows:

- PRoFIT Privacy Manager (PPM). This new component is aimed at enabling the digital witness to follow the PRoFIT methodology [4]. The PPM consists of two subcomponents, the Policy Manager and the Secure Erasure subcomponent. The policy manager handles the group policies defined in [2] plus the new policies to be applied during the PRoFIT phases (c.f. Section 4). The secure erasure module implements the privacy requirement for *use, retention and disclosure limitation* (c.f. Table 1).
- The privacy policies defined by the owner of the digital witness are used as input to the PPM. Thus, the PRoFIT methodology is followed considering the inputs defined by the user. These privacy policies are defined using the OMUD module but they are handled by the policy manager.
- Fine-grain control in the communication with other digital witness and with other modules of the host system. The PPM acts upon the libraries and resources of the host system to make sure that user policies are satisfied (note that a digital witness is a trusted party within a device [2]), but also in the communication with other digital witnesses, to ensure the inclusion of privacy verification in the PRoFIT phases.

The components of the PRoFIT investigator, which are part of the external, remote platform devoted to the analysis of data collected for the investigation, are also included. Moreover, it is important to highlight two types of relationships in the digital witness approach considered in this article:

- Direct relationship between two digital witnesses. DW1 requests the cooperation of DW2 to share information. The data received from DW2 is signed by the owner of DW2.
- Relationship between a digital witness and other entities/devices at the crime scene (see Section 4.1). DW1 requests the data from other devices in the crime scene. These objects are handled by a person in charge, and are pre-configured to encrypt and sign the data to be sent together with the conditions to be satisfied, in order to accept the communication with other devices. The signature of data is done using a secret key derived from the key stored in the digital witness (DW2) of the person in charge, following the binding credentials approach (c.f. Section 2.1).

The following section explains the operation of the digital witness by implementing the operation of the digital witness throughout the phases of a PRoFIT investigation.

## 4. Evaluation

In this section, two use cases within the digital witness approach are shown to illustrate how PRoFIT is applied in the subsequent phases after the preparation of the environment. The first use case presents a malware infection scenario, which shows the flexibility of PRoFIT to properly balance digital forensics and data privacy. The second use case describes a scenario where a search warrant is needed to acquire the digital evidence. In this case, PRoFIT enables a traditional digital evidence collection flow to be followed, that is, it does not take into account privacy preferences, since the cooperation of devices is not required. This use case has been added simply to illustrate that PRoFIT is not restricted to scenarios where it is important to take privacy into account. It can also be used in scenarios where it is paramount to obtain digital evidence as soon as possible regardless of potential negative effects on privacy.

### 4.1. Social Malware

**Figure 6:**
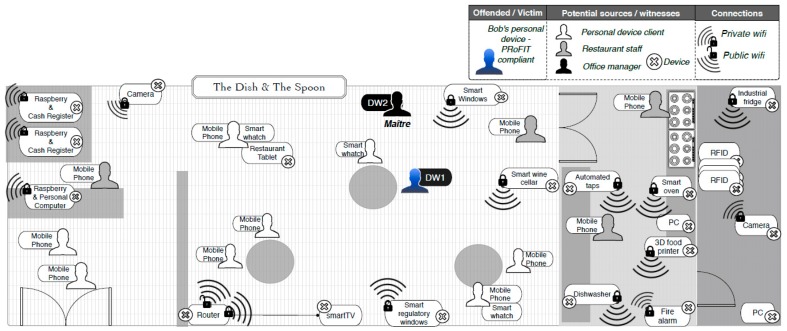
Use case 1: The Dish and The Spoon.

Bob is in possession of a PRoFIT-compliant digital witness (c.f. Section 3.2). He is in a well-known restaurant, *The Dish and The Spoon* (Figure 6), where a number of innovative technologies are used to create exclusive environments, control the supply of ingredients and beverages, increase the security and control the different areas of the restaurant or improve the management of customer requests. In addition, the restaurant offers the *iSpoon* application to its customers for them to get information about their reservations and the theme/ambient they wish for the evening. *iSpoon* uses Bluetooth technology to provide the customer with relevant information (e.g., name of the waiter serving the table, availability of personal, favourite wines, time to be served, etc.). All of this, has made the restaurant one of the most popular in town.

Both personal and non-personal IoT devices coexist in the restaurant. The person in charge of the restaurant’s devices is the the maître (i.e., head waiter and manager). For him it is very important that the devices work correctly so as to guarantee the best experience of the clients during the dinner service.

**Figure 7:**
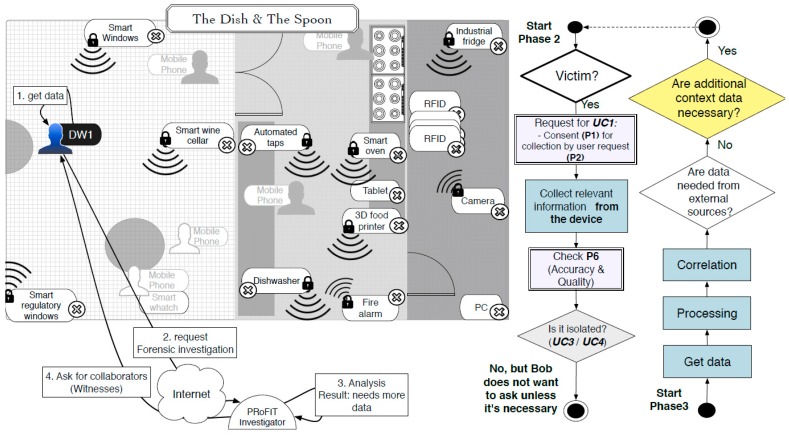
Request the start of digital investigation.

During dinner, Bob’s digital witness (DW1) detects an infection attempt initiated by a device nearby. This provokes the DW to store all the information that might be related to it (e.g., memory dump, network connections, accessed files and running applications) in the last minutes. In addition, the digital witness calculates a cryptographic hash of the digital evidence just collected and alerts Bob, who decides to request an investigation. To this end, Bob sends the evidence stored (and signed) in DW1 to the PRoFIT investigator, thus initiating a digital forensic investigation (phase 2, Figure 7).

The remote system assigns a PRoFIT investigator to the case, who analyses the data provided (phase 3) and confirms that this is a locally-launched attack. Specifically, it looks like a device in the environment is infected and is trying to spread a worm by exploiting a vulnerability in the *iSpoon* application. More concretely, the vulnerability lies in the Bluetooth module used by the application. However, this is not sufficient for the PRoFIT investigator to solve the case, and he/she suggests that DW1 gathers new evidence from other nearby devices willing to collaborate (return to phase 2).

**Figure 8:**
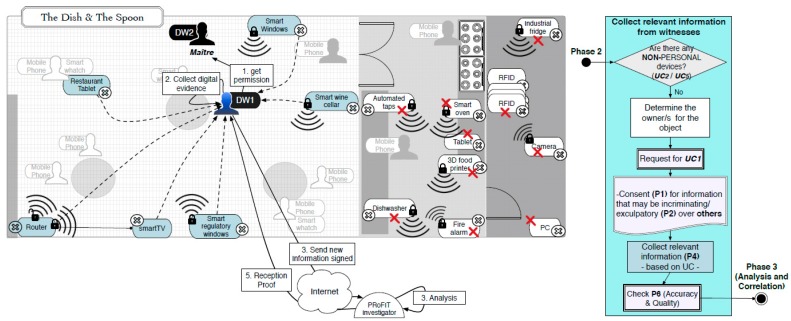
Collect relevant information from witnesses (third parties).

Following the PRoFIT methodology (Section 2.2), DW1 asks non-personal devices first, looking for the person in charge of those devices. In this use case the person in charge of the IoT devices in the room is the maître, who carries another digital witness, DW2. The requester, DW1, initiates a dialogue with the DW2 (denoted external in Figure 5) to obtain the authorisation from the maître to retrieve any data relevant to this particular digital forensic investigation. Figure 8 shows the steps during this part of the process.

When DW1 asks DW2 to collaborate, it attaches some relevant information that can help DW2 decide whether or not to help based on its privacy policies. The collaboration request includes information about the capabilities of Bob’s device and a summary of the preliminary analysis conducted by the PRoFIT investigator. In particular, DW1 certifies that it is a PRoFIT-compliant digital witness, meaning that the platform is based on an anti-tampering core-of-trust and guarantess the privacy of external digital witnesses. The analysis shows evidence that there is a malware threat in the network that spreads using a vulnerability in the Bluetooth.

Once DW2 has checked the credentials sent by DW1 (e.g., credentials and the certificate of the report emitted by the PRoFIT investigator), and after the recommendations provided to the maître, the latter agrees to collaborate but only if the following conditions are met:

- Only remote devices within 100 m from Bob (Bluetooth maximum range is around 100 m.) when the incident occurred may share information.
- However, excluding, in any case, those devices that contain personal data or financial information (e.g., cash registers).
- The shared data will only be used for this investigation and once they are eliminated, the restaurant and also the maître (at that time) will be notified.
- The digital evidence collected will be sent using DW1 as intermediary.

The data provided by the devices of the restaurant are encrypted and signed, and sent to DW1, which, in turn, sends the data to the PRoFIT investigator. In addition, the maître receives a digital receipt that acknowledges that the digital evidence has been sent to the remote PRoFIT investigator. The maître can use this receipt to request that the PRoFIT investigator (i) verifies the contents of the data provided by DW1, and/or (ii) retract and request the deletion of his/her statement. Also, note that the way in which the data is collected depends on the policies defined by the owners or the person in charge of the external digital witness (or collaborators). Therefore, this process can be highly complex depending on the context.

**Figure 9:**
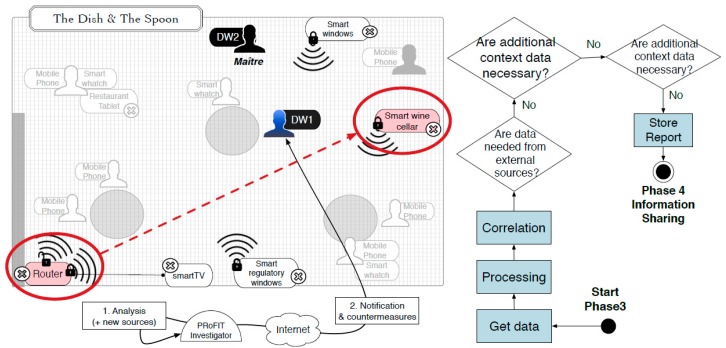
Conclusions of the investigation in the scene.

Once the PRoFIT investigator has received the new digital evidence provided by the maître (phase 3), the results of the investigation suggest that one of the devices in the restaurant (e.g., the smart wine cellar) is infected with the same malware that tried to control Bob’s device (DW1). In fact, the timeline of the events suggests that the attacks received by DW1 came from that device but, before that, it was the router (see Figure 9).

**Figure 10:**
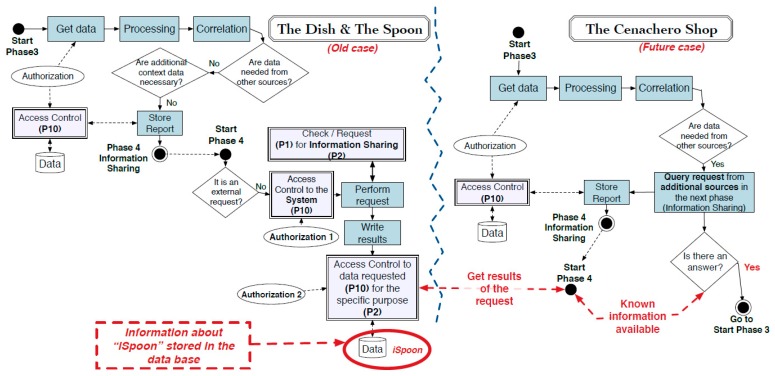
Correlation of information between two separate investigations.

Unfortunately, although the elements involved are determined and it would be possible to stop the infection and minimise the potential damage to other clients, the origin of the cyberattack is not identified. Consequently, the next phase, information sharing (phase 4), is intended to solve this issue. Since Bob is upset because of the attack, he gives his consent for his data to be shared for future forensic investigation. He hopes that doing so will help identify the person responsible for the attack in future cases. Figure 10 shows the information sharing process for this use case following the PRoFIT methodology.

After some time, an improved version of the malware damages other IoT devices. Fortunately, the PRoFIT system kept information about the beginning of the attack in a database (DB), and the malware used, which was called the *iSpoon malware*. These data correlated with other sets of digital evidence from external systems allows the origin of the malware to be determined and a suspect to be arrested. Then, some of the data provided by Bob and other devices are used to prepare the final report (phase 5), which is finally admitted to trial. Finally, the case is settled and some time after the case is closed, the data shared by the cooperators are removed from the PRoFIT system (phase 6).

Although this is a hypothetical scenario and the attack (as well as the application *iSpoon*) are fictitious, it is not unreasonable to think that attacks of this type could occur—or are occurring—without the user even noticing them, as shown in [14].

### 4.2. Warehouse Registration

Police officer Max has to search a warehouse where there are several IoT devices (e.g., cameras, sensors and actuators). It is suspected that some of the devices store digital evidence that may be key to solving an investigation. To help in this task, Max carries a digital witness with capacity of custody, that is, a *digital custodian* [2]. This type of digital witness has some privileges over more usual digital witnesses. In particular, a digital custodian always belongs to an agent of the law. Moreover, in this case, the device stores a signed search warrant and is preconfigured to gather digital evidence relevant to the case (phase 1).

Note that, in this case, the first phase executes both flows shown in Figure 3: the traditional one—following the formal regulatory procedures conducting an official, public investigation—and the preparation of IoT devices in order to acquire digital evidence from the environment as quickly as possible and affecting the integrity of the scenario to the least extent.

During the warehouse registration, Max is the specialist in charge of storing volatile digital evidence using his digital custodian (phase 2). To this end, his device scans the local network and stores the state of the connections and another additional volatile information. It also receives memory dumps and other data that Max decides to store in the digital custodian. All these steps are done obviating the requests and consents of the users because Max has a court order to carry out the aforementioned procedures.

Once in the laboratory, during the analysis (phase 3), the data collected is processed and the relevant digital evidence for the investigation is extracted. In this particular case, no external database queries are required (phase 4). Subsequently, the final reports are written (phase 5), the digital evidence is accepted and the case is ready for its hearing. After some time, the objects collected during the registration, from which the evidence was extracted, are returned to its owner (phase 6).

## 5. Related work

There is a natural tension between computer forensics and privacy (see Table 3 for some examples). However, there are very few papers that focus on this problem and each of them focus more on a specific context (e.g., network forensics versus privacy in [15]).

Most of the approaches that relate these concepts do so from the perspective of (i) analysing the type of data that computer forensics technologies are capable of acquiring (e.g., [16] in mobile phones), (ii) discussing legal compliance [17], or (iii) debating the implications of potentially intrusive monitoring mechanisms for honest users (e.g., [18]). Even a cryptographic model incorporated in a digital investigation framework to protect data privacy is proposed [19]. The solution decrypts suspicious information based on pre-defined keywords.

However, the integration of and balance between privacy and computer forensics is not usual. Table 4 shows the sole solutions that, to the best of our knowledge, deal with this issue in IoT environments. The table compares the accomplishment of privacy principles by these solutions and compares it with the PRoFIT-compliant digital witness.

Themis is an architecture that collects data from sensors in smartphones [20]. The authors argue the need to consider data and user privacy over the entire process. However, this solution is focused on ensuring the acquisition of the data from the end-point devices, but not in promoting the cooperation between devices to obtain such data. Then, although the user is notified, third parties do not have to be notified. Similarly, DroidWatch [21] is a solution for mobile phones, which displays a user consent banner to inform the user about privacy expectations to garner their consent. This is also considered in the initial scheme of digital witness [2], where the users are notified about privacy concerns when they have to choose the type of data that will be stored and reported by their devices. However, in general, current solutions do not consider third-party privacy notification because most of the solutions are not cooperative. This is not erroneous, it just means that they were designed for another purpose. For example, in [22], the authors propose a solution to acquire digital evidence from vehicles that act as witnesses in a vehicular ad-hoc network (VANET). However, privacy principles are not considered, probably because the cost of an incident in such a critical network is sufficient motivation for the adoption of the approach. Moreover, in [1] FEMS is proposed in the context of Home IoT. The authors clarify that privacy in the Home IoT context “*may not necessarily equate to expectations of privacy in social networks*”. The context of a digital witness is pretty similar to a social network in the sense that many individuals can be involved in the same digital investigation. Furthermore, digital witnesses operate in public environments, not just private ones. In addition, alternative IoT-forensic models are analysed in [4].

Precisely due to its characteristics, the PRoFIT model [4] is the one that best fits the digital witness approach, since it considers the use of devices prepared to acquire electronic evidence and the cooperation of the entities, and also privacy principles are included in the core of the methodology. Therefore, when adapting the digital witness approach to meet the requirements of the PRoFIT model, a solution is obtained that balances digital forensics and privacy requirements in the context of a digital investigation which depends on the cooperation of the devices in the environment.

## 6. Conclusions and Future Work

Computer forensics and privacy are two confronting disciplines. Typically, these confrontations are solved by applying the law to guarantee the admissibility of electronic evidence and the fundamental right to privacy of the individuals. However, this is not enough for cooperative IoT scenarios, where the joint application of privacy mechanisms together with computer forensics mechanisms can help to obtain crucial data to understand the context of a cybercrime and settle an investigation. In this paper, a solution to collect digital evidence from IoT environments called *digital witness* has been adapted to support the 11 privacy principles considered in the various phases of the PRoFIT methodology. As a consequence of this synergy, an IoT-Forensics solution integrates privacy requirements and mechanisms as part of its design thus encouraging the voluntary cooperation of digital witnesses in IoT scenarios. The approach has been validated through the definition of use cases with and without privacy requirements, which shows that the solution is capable of finding a balance between privacy and IoT-Forensics principles.

Future work is aimed at the formal definition of privacy policies for digital evidence collected from personal devices, considering different user profiles, resources and functionalities in the devices. Furthermore, the paper is not intended to give all the technical details on how to implement the methodology into a specific hardware. Indeed, there is no single way of implementing the methodology and this is a matter of current and future work.

## Figures and Tables

**Table 1:** Privacy principles in PRoFIT.

ISO/IEC 29100	PRoFIT Phases					
	PP	CC	AC	IS	PT	RV
1. Consent and choice	✓	✓		✓		
2. Purpose legitimacy and specification	✓	✓		✓		
3. Collection limitation		✓				
4. Data Minimisation	✓				✓	
5. Use, rentention and disclosure limitation						✓
6. Accuracy and quality		✓			✓	
7. Openness, transparency and notice	✓					✓
8. Individual participation and access		✓				
9. Accountability			✓			
10. Information security controls			✓	✓		
11. Compliance	✓	✓	✓	✓	✓	✓

PP. Preparation, CC. Context-based collection, AC. Analysis and correlation, IS. Information sharing, PT. Presentation, RV. Review.

**Table 2:** Mapping of privacy risks and mitigation mechanisms in a PRoFIT-compliant DW.

Privacy Risk for DW	Mitigation Mechanisms	PRoFIT Phases					
		PP	CC	AC	IS	PT	RV
Devices nearby may know when a DW has been disabled from its duties	Direct Anonymous Attestation [8]	✓	✓				
Acknowledge the acquisition of digital evidence without the signer accessing the contents	Blind signatures + signature chaining [9]	✓	✓				
Witnesses may be reluctant to share their own version of the incident with other participants	Homomorphic encryption or secure computation [10]	✓	✓				
The identity of a *witness* is known	Anonymous digital witnessing [3]	✓	✓	✓			
The identity of those involved in the discovery process is exposed	Anonymous route discovery [11]		✓	✓			
The system could expose other users as being part of the environment	Third-party user consents		✓	✓	✓	✓	✓
Transactions could show private information	Privacy-aware smart contracts [12]		✓		✓		✓
The data shared with an investigator could be used for additional purposes without consent	Disposal Guarantees [13]		✓				✓

PP. Preparation, CC. Context-based collection, AC. Analysis and correlation, IS. Information sharing, PT. Presentation, RV. Review.

**Table 3:** Similarities and Conflicts between Digital Forensics and Privacy.

Example	Privacy	Computer Forensics
Onion routing	Privacy in communications (e.g., Tor)	Affects traceability
Anonymity	Hides the identity of the individual	Affects liability and traceability
Data encryption	Confidentiality, data privacy	Makes data analysis difficult/impossible.
Aggregation	Data minimisation	Relevant data can be lost and traceability affected
Secure erasure	Data privacy	Lost of digital evidence
Report incidence	Affects location privacy and anonymity if the subject’s identity is indicated	Adds value to data correlation and verification.
Data collection	Can provide sensitive information about the environment	Allows to obtain more verifiable information
Data correlation	Affects linkability; can help to get information about the identity of third parties (and other data)	Can help to deduce new relevant information for the case
Node discovery	Affects location privacy	Potential sources of data
Legal procedures	Privacy as humans right	Admissibility of digital evidence

Gray: potential disadvantage. White: potential advantage.

**Table 4:** Related work that considers privacy.

ISO/IEC 29100:2011	Proactive IoT-Forensic Solutions			
	Themis	DroidWatch	DW	PRoFIT-Compliant DW
1. Consent and choice	✓(user)	✓(user)	✓(user)	✓(user & third party)
2. Purpose legitimacy and specification		✓(user)		✓(user & third party)
3. Collection limitation				✓
4. Data Minimisation				✓
5. Use, rentention and disclosure limitation				✓
6. Accuracy and quality	✓			✓
7. Openness, transparency and notice				✓
8. Individual participation and access				✓
9. Accountability				✓
10. Information security controls	✓		✓	✓
11. Compliance				✓
